# Exploring visual pain trajectories in neck pain patients, using clinical course, SMS-based patterns, and patient characteristics: a cohort study

**DOI:** 10.1186/s12998-022-00443-3

**Published:** 2022-09-08

**Authors:** Pernille Irgens, Birgitte Lawaetz Myhrvold, Alice Kongsted, Bård Natvig, Nina Køpke Vøllestad, Hilde Stendal Robinson

**Affiliations:** 1 Department of Interdisciplinary Health Sciences, Institute of Health and Society, University of Oslo, Blindern, P.O. Box 1089, 0317 Oslo, Norway; 2 Department of Sports Science and Clinical Biomechanics, University of Southern Denmark, Odense, Denmark; 3 Chiropractic Knowledge Hub, Odense M, Denmark; 4 Department of General Practice, Institute of Health and Society, University of Oslo, Oslo, Norway

**Keywords:** Visual trajectories, Longitudinal, Subgrouping, Recall bias, Chiropractic, SMS, Questionnaire

## Abstract

**Background:**

The dynamic nature of neck pain has so far been identified through longitudinal studies with frequent measures, a method which is time-consuming and impractical. Pictures illustrating different courses of pain may be an alternative solution, usable in both clinical work and research, but it is unknown how well they capture the clinical course. The aim of this study was to explore and describe self-reported visual trajectories in terms of details of patients’ prospectively reported clinical course, their SMS-based pattern classification of neck pain, and patient’s characteristics.

**Methods:**

Prospective cohort study including 888 neck pain patients from chiropractic practice, responding to weekly SMS-questions about pain intensity for 1 year from 2015 to 2017. Patients were classified into one of three clinical course patterns using definitions based on previously published descriptors. At 1-year follow-up, patients selected a visual trajectory that best represented their retrospective 1-year course of pain: single episode, episodic, mild ongoing, fluctuating and severe ongoing.

**Results:**

The visual trajectories generally resembled the 1-year clinical course characteristics on group level, but there were large individual variations. Patients selecting Episodic and Mild ongoing visual trajectories were similar on most parameters. The visual trajectories generally resembled more the clinical course of the last quarter.

**Discussion:**

The visual trajectories reflected the descriptors of the clinical course of pain captured by weekly SMS measures on a group level and formed groups of patients that differed on symptoms and characteristics. However, there were large variations in symptoms and characteristics within, as well as overlap between, each visual trajectory. In particular, patients with mild pain seemed predisposed to recall bias. Although the visual trajectories and SMS-based classifications appear related, visual trajectories likely capture more elements of the pain experience than just the course of pain. Therefore, they cannot be seen as a proxy for SMS-tracking of pain over 1 year.

**Supplementary Information:**

The online version contains supplementary material available at 10.1186/s12998-022-00443-3.

## Background

Non-specific neck pain is costly and common [1–3]. Close to one third of all adults are likely to experience neck pain during 1 year [4]. During the last years, considerable research on spinal pain has focused on subgrouping patients based on prognostic factors and individual clinical courses of pain [5–9]. Categorizing pain based on the temporal variation as either persistently fluctuating or episodic seems to have replaced the more traditional categories of chronic and acute pain [5, 10–12]. Furthermore, common pain trajectories have been established for low back pain [5], and are also found in neck pain [13–16]. Definitions and terminology of trajectories for low back pain have been translated into subgroup criteria [17], which fit readily to neck pain patients [13].

Trajectories appear to be stable over time [18, 19], as well as representing different patient profiles across various health domains [5, 13, 15]. Hence, it is likely they are better measures in clinical studies than single pain measures at single time-points. They may also be useful as a stratification tool, or as a tool in clinical management and communication. However, identifying accurate pain trajectories is time-consuming, expensive, and not feasible in clinic or most research, and methodological quality (reliability and validity) is still unknown.

A recent study on low back pain has introduced a novel and simple alternative to long-term follow-ups with frequent measurements to identify clinical course, namely, to use pictures illustrating the different pain trajectories (visual trajectories) [20]. Patients were asked to choose the picture that best represented their clinical course of pain (trajectory) from among eight illustrations. Patients and clinicians easily identified with the visual trajectories, indicating good face validity. This method is straightforward, quick, and cheap to administer, and therefore probably more easily applicable in clinical practice. Similar visual trajectories were recently found to slightly improve a clinical prediction rule for neck pain [21]. We have recently shown that classification of patients based on visual trajectories reflected group differences in severity regarding symptoms and distress [22].

To our knowledge, no study has explored the association between SMS-based and visual trajectories in neck pain patients. While SMS-based trajectories describe the prospectively reported course of pain, visual trajectories provide the patients' retrospective perception of the course. Visual trajectories may represent anything from a recall that is largely disconnected from the experienced course, to a recall that closely resembles the patient’s SMS-based trajectory. For visual trajectories to be useful in research and clinic, it is essential to understand what they capture regarding the clinical course from prospective frequent measures. Thus, the aim of this study was to explore and describe self-reported visual trajectories in terms of details of patients’ prospectively reported clinical course, their SMS-based pattern classification of neck pain, and patients’ characteristics.

## Method

### Study design, population and setting

We used data from a 1-year observational, multi-center, practice-based cohort consisting of patients with neck pain in a chiropractic care setting in Norway. Seventy-one chiropractors located across Norway invited eligible patients with neck pain to participate in the study between September 2015 and June 2016. The chiropractors provided written and verbal information to patients interested in participating. Patients accepting to participate signed a written consent form. Decisions regarding treatment and follow-up were at the chiropractors' discretion, and unaffected by participation in the study. Descriptions of cohort recruitment and study procedures are published in a previous paper [13]. The Regional Committee for Medical and Health Research Ethics (2015/89) approved the study protocol. The study was reported according to the STROBE statement [23].

#### Population

**Fig. 1:**
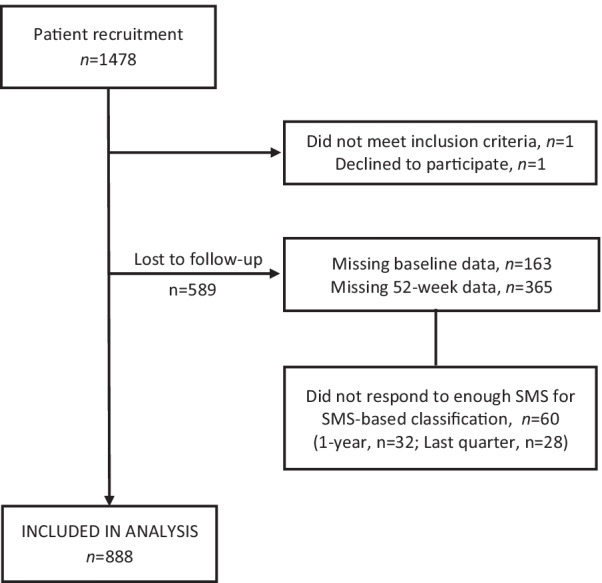
Flow-chart of study population

We included patients aged 18 years or more, presenting with bothersome neck pain as their primary or secondary complaint, independent of being acute or long-term or in a treatment plan. Patients had to have basic Norwegian reading and writing skills and be able to operate a mobile phone. We excluded patients with suspected inflammatory diseases, fractures, systemic pathology, or nerve root involvement requiring referral to surgery. The chiropractors recruited 1478 patients with neck pain. Of these, 888 (60%) had completed both 1-year and baseline questionnaires and provided enough SMS responses to be classified to an SMS-based pattern, and thus, constituted the study sample (Fig. 1).

### Data collection

Patients received questionnaires electronically or on paper. Paper questionnaire was given by the chiropractor at recruitment. For patients selecting electronic questionnaire, the chiropractor gave the patient’s e-mail address to the research group, who sent an e-mail to the patient with a link to the questionnaire within 2 days after recruitment. We collected questionnaire data at baseline, 4 weeks, 12 weeks, and 1-year follow-up, but the present study used questionnaire data from baseline and 1-year follow-up. Patients not responding within 7 days received one written reminder, followed by a phone call 2 weeks later. Patients also received 2–3 mobile text messages (SMS) at the same day and time every week over a 1-year period, with a reminder 2 days later should they fail to respond to the initial SMS. We collected the following patient demographics at baseline: age, sex, history of neck pain and consultation type, as well as pain intensity at recruitment. History of neck pain was categorized into those with a history of neck pain less than 5 years, and those with equal to, or more than, 5 years history. We defined patients recruited at their first visit for a new episode of neck pain as "first consultation".

#### 1-Year questionnaire data

We measured current neck pain intensity on a 0–10 Numeric Rating Scale (NRS) (0 = no pain; 10 = worst pain imagined) [24]. Functional status was measured by the Neck Disability Index (NDI). The NDI consists of 10 items regarding pain and function with scoring from 0 to 5. The sum score ranges from 0 to 50 points, with higher scores indicating more disability [25, 26]. We measured emotional stress by the Hopkins Symptom Checklist (HSCL-10), with scoring from 1 (low) to 4 (high) [27, 28], and psychosocial risk factors by the Örebro Musculoskeletal Pain Questionnaire [29, 30]. The Örebro sum score ranges from 0 to 100 points, where higher scores indicate higher risk of persistent pain and disability. Studies have shown that expectations are partly, but not completely, formed by pain history [31, 32]. We therefore measured recovery expectations from Item 7 of the Örebro screening questionnaire [33], “In your view, how large is the risk that your current pain may become persistent?” (0–10, 0 = no risk, 10 = very large risk). We also recorded characteristics of symptoms regarding duration of the current episode (< 1 month, 1–3 months, > 3 months) and pain radiating into the shoulder and/or the elbow (yes/no). Number of pain sites was measured by the Nordic pain questionnaire [NPQ (0–10)] [34]. We used functional status (NDI), emotional stress (HSLC-10), psychological risk factors (Örebro screening questionnaire), and recovery expectations [33] to calculate change in the relevant scores between baseline and 1-year follow-up. As there is uncertainty about the concept and measurement of minimal important change (MIC) [35], we decided to calculate the patients’ change in scores as follows: We subtracted the baseline score from the 1-year score. Patients with a change score equal to or higher than the 80th percentile score for the whole cohort were defined as having a positive change.

#### Visual trajectories

**Fig. 2:**
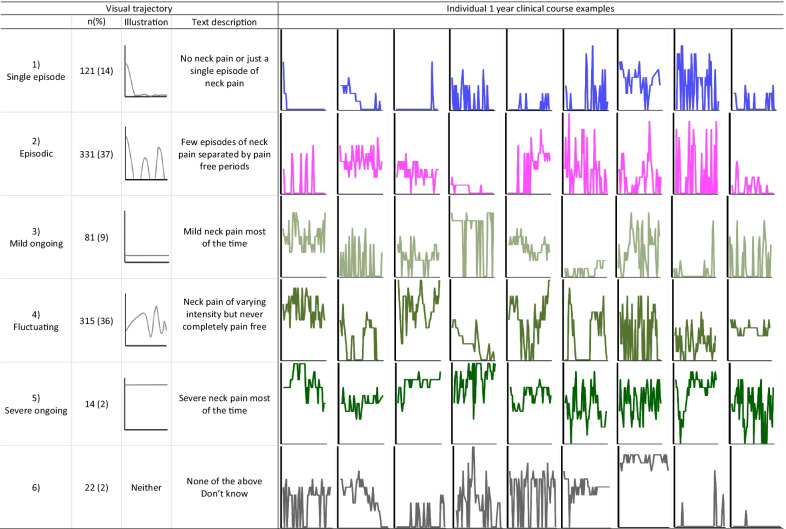
Description of the visual trajectories and examples of 1-year individual SMS-based clinical course

In the 1-year questionnaire, we asked patients to identify their neck pain course over the previous year, using a self-reported visual trajectory pattern questionnaire developed for LBP [5] (hereafter ‘visual trajectory’). The questionnaire has previously been used in two studies from our NP cohort [21, 22]. It includes drawings and descriptions of five different pain trajectories; No neck pain or Single episode (hereafter ‘Single episode’), Episodic, Mild ongoing, Fluctuating, and Severe ongoing, with the corresponding question: “Please tick off the description below that you think best represents how your neck pain has been the previous 12 months” (Fig. 2). The questionnaire also included the answer alternatives “None of the above” and “Do not know”.

#### Clinical course from SMS data

Patients received the following questions weekly via SMS: “How many days the last week has your neck been bothersome? Please answer with a number between 0 and 7” (hereafter ‘paindays’). If the answer was between 1 and 7, the patient received a second SMS: “How intense has your neck pain typically been the last week? 0 = no bother, 10 = worst possible bother” (hereafter ‘pain intensity’). A third SMS (not used in the present study): “How many days this last week has your neck limited your daily activities? Please answer with a number between 0 and 7”.

For the descriptors of the course of pain, we calculated the total number of paindays, the mean pain intensity across the 52 weeks, the duration and frequency of pain-free and painful weeks, as well as the proportion of the weeks that were pain-free, in the minor, mild, moderate, and severe pain range (defined below) for each patient. As a measure of variation in pain intensity within individuals, we calculated the standard deviation (SD) of the mean of the individual’s weekly pain intensity (1-year and the last quarter) (hereafter ‘intensity variation’).

##### Classification into SMS-based patterns

We described the patients’ clinical course, using the same criteria as in recently published articles [13, 17, 19]. Patients were classified into patterns based on pain intensity from the weekly SMS data collected over 1 year (hereafter ‘SMS-based pattern’). The predefined SMS-based patterns included four variation patterns: Persistent fluctuating, Episodic, Single episode and Recovery. In the Persistent fluctuating pattern, no pain-free period could last 4 weeks or longer. Patients in the Episodic pattern must have at least one pain-free period of minimum four consecutive weeks between weeks with pain. The pain-free duration was based on consensus-based definitions [36, 37], and has been tested in a low back pain cohort [38]. The Single episode was defined as a short flare-up lasting 1–2 weeks anywhere during the study period. The Recovery pattern included all patients with maximum pain intensity < 2. We subsequently split the Persistent fluctuating pattern into four subgroups based on mean pain intensity as follows: Severe (pain intensity ≥ 6), Moderate (4 ≤ pain intensity < 6), Mild (2 ≤ pain intensity < 4), and Minor (pain intensity < 2). This is in line with previously suggested cut-off values for pain [39–42]. We split the Episodic and Single Episode patterns into three subgroups each, based on the maximum pain intensity reported throughout the period: Severe (pain intensity ≥ 6), Moderate (4 ≤ pain intensity < 6) and Mild (2 ≤ pain intensity < 4). We combined the Recovery pattern and the Single episode pattern into one pattern called “Single episode/Recovery”. This left us with 3 patterns and 11 subgroups for analyses. The process of reducing the number of subgroups from the original 16 [17] to the 11 used in this study is described in Additional file 1.

We have previously found that patients in an episodic pain course have large individual and group variations in painful- and pain-free periods [19]. We therefore wanted to explore the relationship between the stability of the patients’ SMS-based pattern over 1 year and their selected visual trajectory. We therefore used the above-mentioned classification procedure on data from two shorter periods: the first and the last quarter (weeks 1–13 and weeks 40–52, respectively) of the follow-up year. We defined patients allocated to the same pattern in the first and last quarter as having a stable trajectory, and those with different patterns as having a shifting trajectory, as done previously [19]. We calculated the proportion of patients that had a stable pattern.

### Data analyses

Few of our variables were normally distributed and accordingly we present descriptive variables as frequencies and percentages or median with interquartile range (IQR) when appropriate. We combined the visual trajectory alternatives “None of the above” and “Do not know” into one group, called “Neither” for analytical purposes. The methods used for imputing the missing values on the weekly pain intensity measures for the SMS-based pattern is described in detail in Additional file 1. Patients who had provided 26 or more responses for the full year (52 weeks) and 6 or more responses for the last quarter (13 weeks) after imputation were included in the analyses (Fig. 1).

We cross-tabulated visual trajectories with the eleven SMS-based patterns to explore and describe the distribution of SMS-based pattern classifications for each of the visual trajectories. We present the distribution of SMS-based patterns as a stacked bar graph for each of the visual trajectories at 1-year follow-up. To explore pain recall in relation to the selection of visual trajectory, we did the same cross-tabulation and stacked bar graph presentation between the visual trajectories and the last quarter SMS-based patterns.

This study was part of a larger project aiming to identify prognostic factors for neck pain. The larger project aimed to assess 11 possible prognostic factors. The number of participants needed was calculated as follows: 10 patients per prognostic factor is required in multiple regression models gives n = 1100 [43]. Expecting a possible 20% drop-out, we needed a sample size of 1320 patients for the prognostic study. As the present study did not include hypothesis testing, no new sample size calculation was performed. However, we needed a sample size allowing a reasonable description of each of the visual trajectories, which we expected to include at least 5% of the sample.

We carried out all analyses using STATA 16 (StataCorp, Texas, USA).

## Results

The 888 patients had a mean age of 45 (SD 13) years and 663 (75%) were women. The mean pain intensity (SD) at baseline and 1-year follow-up was 4.1 (2.3) and 2.5 (2.4) respectively. Close to 50% reported previous neck pain duration of 1 year or longer at baseline. There were no substantial differences between the study sample and those lost to follow-up. For further details of cohort characteristics, see Table 1.

**Table 1:** Clinical course details and characteristics of patients presented for the cohort and each of the visual trajectories (n = 888)

	Cohort	Single episode	Episodic	Mild Ongoing	Fluctuating	Severe Ongoing	Neither
Number, n (%)	888 (100)	121 (14)	331 (37)	82 (9)	318 (36)	14 (2)	22 (2)
*Weekly SMS-based details*							
Total number of days with pain, median (IQR)	100 (51–175)	26 (8–50)	71 (44–110)	114 (70–166)	180 (129–264)	315 (267–331)	74 (23–139)
Proportion (%) of weeks, median (IQR)							
No or minor pain (< 2)	33 (4–65)	83 (67–94)	49 (26–71)	26 (3–60)	4 (0–24)	0 (0–2)	31 (4–79)
Mild pain (≥ 2 < 4)	24 (12–40)	10 (2–24)	25 (16–40)	38 (20–59)	26 (12–41)	7 (0–20)	19 (2–35)
Moderate pain (≥ 4 < 6)	18 (6–32)	4 (0–8)	12 (6–25)	17 (4–28)	29 (19–44)	25 (12–35)	20 (4–37)
Severe pain intensity (≥ 6)	4 (0–16)	0 (0–2)	4 (0–10)	2 (0–8)	17 (4–37)	62 (27–86)	6 (0–17)
Mean pain intensity, median (IQR)^a^	4 (2–6)	0.5 (0.2–1.1)	1.8 (1.1–2.6)	2.3 (1.4–3.2)	3.8 (2.8–4.9)	6.1 (4.5–7.5)	2.5 (0.7–3.6)
Mean duration of pain-free periods (weeks), median (IQR)	2 (1–8)	9 (5–23)	3 (2–4)	2 (1–3)	2 (1–3)	0 (0–0)	2 (2–11)
Longest pain-free period (weeks), median (IQR)	6 (2–13)	22 (10–38)	6 (3–11)	3 (1–7)	1 (0–1)	0 (0–0)	5 (1–15)
No pain last four weeks, %	20	64	23	9	3	0	23
Number of painful periods, median (IQR)	4 (1–6)	3 (1–8)	7 (5–9)	5 (4–8)	1 (1–5)	1 (1–1)	4 (1–9)
Longest painful period (weeks), median (IQR)	11 (6–22)	4 (2–9)	9 (5–15)	12 (7–21)	20 (10–52)	39 (10–52)	9.5 (4–16)
Pain intensity variation 1 year^b^, median (IQR)	1.2 (1.1–2.0)	1.1 (0.7–1.5)	1.7 (1.3–2.1)	1.3 (1.0–1.8)	1.6 (1.2–2.0)	1.7 (1.0–2.0)	1.7 (1.1–2.0)
Pain intensity variation last quarter^b^, median (IQR)	1.2 (0.8–1.7)	0.3 (0.0–1.1)	1.5 (0.9–2.0)	0.9 (0.6–1.4)	1.3 (0.9–1.8)	1.0 (0.8–1.7)	1.3 (0.6–1.7)
Stable SMS-based pattern, yes %	71	38	62	77	91	100	68
*1-year questionnaire data*							
Pain intensity, median (IQR)	2 (0–4)	0 (0–0)	1 (0–3)	2 (1–4)	4 (3–5)	6 (5–8)	0.5 (0–3)
Duration of episode < 1 month, %	15	46	17	10	3	0	19
Radiating pain to shoulder/elbow, %	64	34	50	76	86	92	36
Recovery expectations, median (IQR)	6 (3–9)	0 (0–2)	5 (2–8)	6 (4–9)	9 (7–10)	10 (8–10)	4 (1–7)
Örebro screening questionnaire, median (IQR)	34 (23–46)	17 (11–23)	30 (22–38)	32 (23–40)	46 (37–54)	60 (49–65)	26 (14–44)
HSCL-10, median (IQR)	1.4 (1.2–1.8)	1.1 (1.0–1.4)	1.4 (1.2–1.7)	1.4 (1.2–1.7)	1.6 (1.3–2.0)	2.0 (1.5–2.2)	1.2 (1.0–1.5)
NDI, median (IQR)	9 (4–14)	2 (1–4)	7 (4–10)	7 (5–11)	14 (10–18)	23 (18–30)	5 (2–11)
Number of MSK pain sites, median (IQR)	4 (2–6)	2 (0–3)	3 (2–5)	4 (2–6)	5 (4–7)	7 (4–8)	3 (2–5)
Health status, (median IQR)	80 (63–90)	90 (80–95)	80 (70–90)	80 (75–88)	70 (50–80)	50 (30–64)	82.5 (70–90)
*Baseline patient characteristics*							
Age (y), median (IQR)	45 (14)	45 (35–53)	43 (35–52)	47 (38–54)	46 (38–55)	46 (41–63)	46 (35–57)
Female, %	655 (74)	60	78	63	79	57	68
First consultation, %	135 (16)	21	14	14	14	15	32
Physical activity, yes %	69	69	68	70	70	57	77
Pain intensity, median (IQR)	4 (2–6)	3 (0–4)	4 (2–5)	3 (2–5)	5 (4–6)	5 (5–7)	4 (2–6)
No previous episodes, %	13	33	13	10	7	14	14
NP history baseline > 5 years, yes %	69	48	71	64	75	92	47

Pain intensity from NRS: the 11-point numerical rating scale [24], Recovery expectations from “In your view, how large is the risk that your current pain may become persistent?” (0–10, 0 = no risk, 10 = very large risk) and psychosocial risk factors: Örebro Screening Questionnaire (0–100) [29, 33], HSCL-10: Hopkins Symptom Checklist measuring emotional stress (0–4) [28], NDI: Neck Disability Index (0–50) measuring disability [26], Health status (0–100) [58], NP: Neck pain

^a^Presented as median of individual mean pain intensity

^b^Presented as standard deviation from individual mean pain intensity

In total, 37% (n = 331) of patients selected the Episodic visual trajectory, 36% (n = 318) the Fluctuating trajectory, and 14% (n = 121) the Single episode trajectory on the visual trajectory questionnaire. Furthermore, 9% (n = 82) selected Mild Ongoing and 2% (n = 14) selected the Severe Ongoing trajectory. Two percent of patients (n = 22) did not identify with any of the five visual trajectories. Using the SMS-based classification, 48% of the study sample were classified as Persistent fluctuating and 49% as Episodic. Examples of individual SMS-based trajectories for each of the visual trajectories are displayed in Fig. 2. These examples are selected to illustrate the variability of the individual clinical courses of pain.

### Clinical course characteristics

**Fig. 3:**
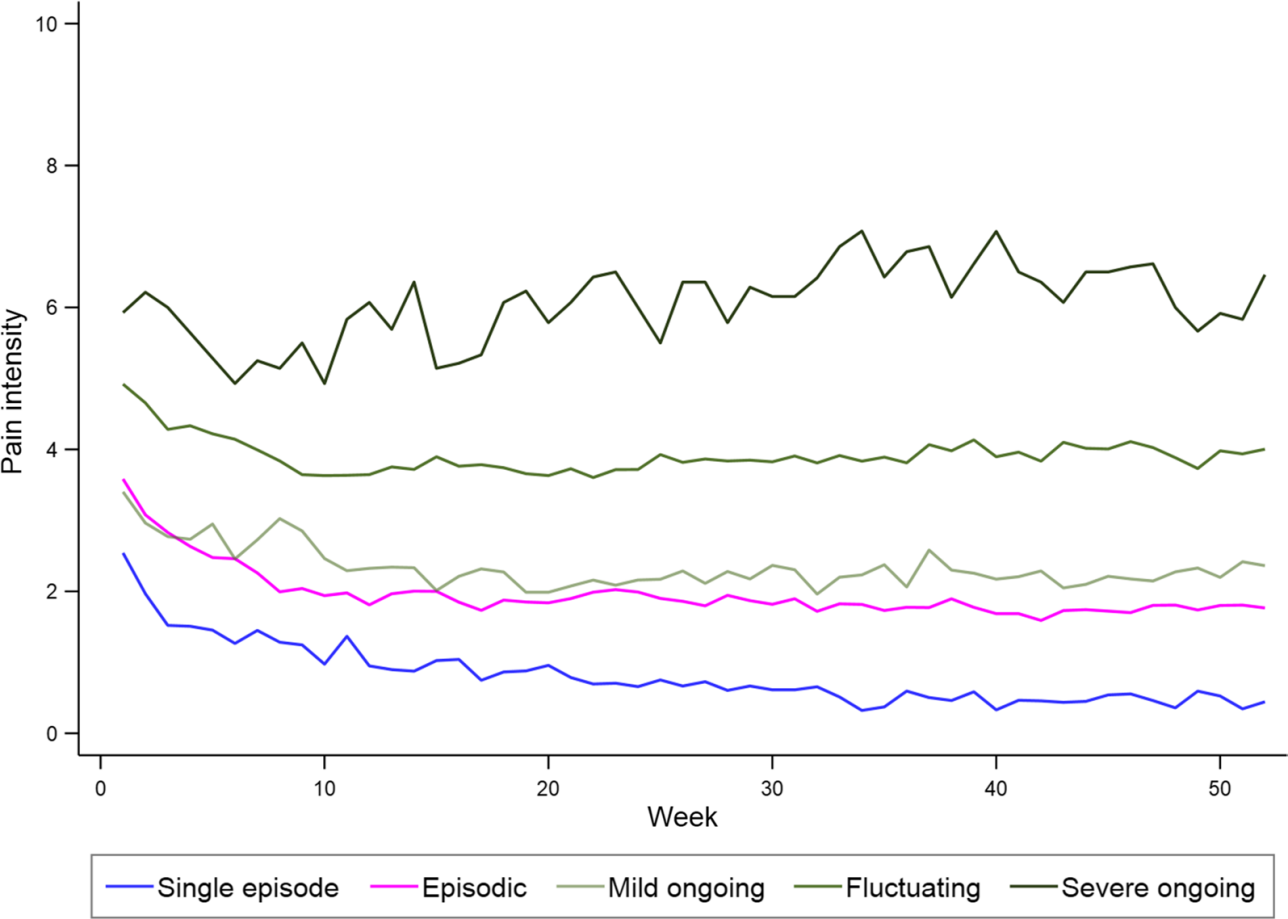
Weekly mean pain intensity over 1 year in the five visual trajectories

The details of the clinical course varied largely among patients selecting the same visual trajectory. However, there were clear differences between the different visual trajectories concerning the mean course of pain and the descriptors of the clinical course for all, except between the episodic and mild ongoing visual trajectories (Fig. 3 and Table 1).

In general, the visual trajectory resembled well the predefined clinical course descriptors on a group level. For instance, patients selecting the Single episode visual trajectory were likely to have the highest proportion of pain-free weeks (median 83%, Inter-Quartile Range (IQR) 67–94%) with only short periods with pain, and they rarely or never reported painful periods with moderate or severe pain (Table 1). However, they reported large variations concerning the number of painful periods during the 1-year follow-up (median 3, IQR 1–8). Patients selecting a Fluctuating visual trajectory were likely to have minimal numbers of pain-free weeks, report moderate to high pain intensity most weeks, but the weekly variation in pain were similar to the patients selecting the Episodic visual trajectory. Similarly, patients selecting the Severe ongoing visual trajectory have variations in weekly pain, but they report the highest pain intensity, most days with pain, and no pain-free weeks. Patients selecting Episodic or Mild ongoing visual trajectories have a clinical course in between those selecting the Single episode and Fluctuating trajectories, namely frequent pain episodes with mostly minor or mild pain. The mean course of pain differed for each of the visual trajectories apart from Episodic and Mild ongoing, which again were very similar (Fig. 3). Although the visual trajectories are generally different, there was a large overlap in the detailed course for the patients selecting them, as seen from IQRs in Table 1, especially between those selecting the Episodic and Mild ongoing visual trajectories.

### Associations between visual trajectories and classification into SMS-based patterns

**Fig. 4:**
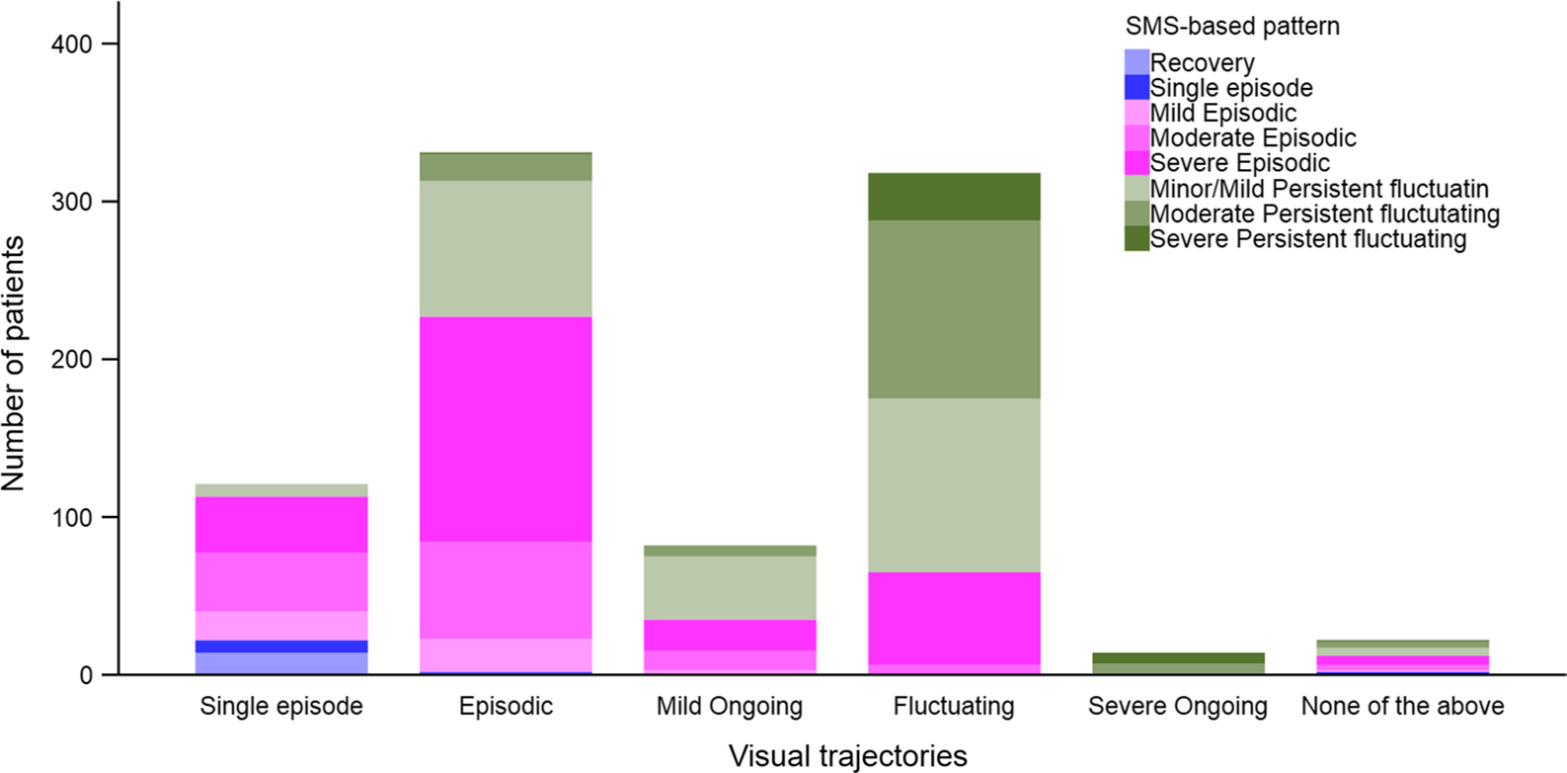
Relationship between the five visual trajectories and the 1-year SMS-based patterns

Figure 4 shows the frequency of the SMS-based patterns for each of the visual trajectories (for details see Additional file 1: Table S2). The majority (75%) of the patients selecting a Single episode visual trajectory were classified as Episodic and 18% as Single episode/Recovery. Sixty-eight percent of patients selecting an Episodic visual trajectory were classified as Episodic, with most of the remaining (31%) classified as Minor to Moderate Persistent fluctuating. For patients selecting the Mild ongoing visual trajectory, 49% were classified Mild or Minor Persistent fluctuating, and 39% were classified as Severe or Moderate Episodic. The majority of patients selecting a Fluctuating or Severe ongoing visual trajectory were classified as Persistent fluctuating (80% and 100%, respectively) and 19% of those selecting Fluctuating pattern were classified as Severe Episodic. None of the patients selecting the Mild ongoing, Fluctuating or Severe ongoing trajectories were classified as Single episode/Recovery on SMS.

### 1-Year questionnaire data

The data reported for symptoms, disability and psychosocial factors showed an increase in severity from those selecting the Single episode visual trajectory to the Severe Ongoing visual trajectory (Table 1). In addition, the majority of patients selecting the Ongoing or Fluctuating visual trajectories had a stable SMS-based pattern. Patients selecting Episodic or Mild ongoing visual trajectories and classified as Episodic with SMS data were almost similar in both clinical course and patient characteristics (Table 2). This group was different from patients selecting Single episode visual trajectory and classified as Episodic (less pain and less bothered) and patients selecting Fluctuating visual trajectory and classified as Episodic (more pain and more bothered). Similar associations were found in patients selecting Episodic or Mild ongoing visual trajectory but classified as Persistent fluctuating. These patients had less pain-free weeks, higher pain intensity and longer painful periods compared to patients selecting Episodic or Mild ongoing visual trajectory and classified as Episodic.

**Table 2:** Weekly SMS-based details and patient characteristics by patients’ selected visual trajectNumber, nory and their 1-year classified Episodic or Persistent fluctuating pattern (n = 888)

	Visual trajectory				Visual trajectory		
	Episodic SMS-based pattern				Persistent fluctuating SMS-based pattern		
	Single episode	Episodic	Mild ongoing	Fluctuating	Episodic	Mild ongoing	Fluctuating
Number, n	91	225	35	65	104	47	253
*Weekly SMS-based details*							
Total number of days with pain, median (IQR)	100 (81–118)	122 (103–146)	134 (112–172)	154 (130–208)	183 (154–210)	211 (176–268)	273 (221–352)
Proportion (%) of weeks, median (IQR)							
Minor pain (< 2)	80 (67–90)	61 (46–78)	60 (43–76)	44 (33–56)	17 (8–29)	10 (0–24)	2 (0–8)
Mild pain (≥ 2 < 4)	13 (6–24)	21 (13–31)	21 (13–39)	22 (14–33)	41 (28–55)	52 (33–71)	29 (12–44)
Moderate pain (≥ 4 < 6)	4 (2–8)	8 (4–20)	8 (2–16)	17 (12–25)	25 (14–37)	25 (13–37)	35 (23–47)
Severe pain intensity (≥ 6)	0 (0–2)	2 (0–6)	2 (0–4)	6 (4–19)	8 (2–16)	2 (0–8)	19 (6–41)
Mean pain intensity, median (IQR)^a^	0.6 (0.3–1.1)	1.4 (0.9–2.0)	1.4 (0.9–1.9)	2.1 (1.6–3.0)	2.7 (2.3–3.4)	3.0 (2.4–3.5)	4.3 (3.4–5.1)
Longest pain-free period (weeks), median (IQR)	19 (10–28)	8 (5–14)	7 (5–13)	7 (5–9)	2 (1–2)	1 (0–2)	0 (0–1)
No pain last four weeks, %	67	33	20	15	0	0	0
Number of pain-free periods, median (IQR)	1 (0–2)	2 (1–3)	1 (1–2)	2 (1–3)	1 (0–2)	0 (0–1)	0 (0–0)
Longest painful period (weeks), median (IQR)	4 (3–9)	7 (4–11)	9 (4–12)	9 (6–14)	16 (10–28)	16 (10–39)	26 (13–50)
Stable SMS-based pattern, yes %	25	44	46	58	99	100	100
*1-year patient characteristics*							
Pain intensity, median (IQR)	0 (0–0)	0 (0–2)	1 (0–3)	3 (0–5)	3 (2–5)	3 (2–4)	4 (3–6)
Recovery expectations, median (IQR)	1 (0–2)	4 (2–7)	5 (2–8)	8 (5–10)	7 (4.5–9)	7 (5–10)	9 (7–10)
Örebro screening questionnaire, median (IQR)	17 (11–25)	27 (19–34)	27 (21–39)	37 (30–49)	35 (28–44)	34 (26–40)	47 (40–55)
HSCL-10, median (IQR)	1.1 (1.0–1.5)	1.3 (1.1–1.6)	1.3 (1.1–1.5)	1.4 (1.2–1.8)	1.5 (1.2–1.7)	1.5 (1.2–1.9)	1.6 (1.3–2.0)
NDI, median (IQR)	2 (1–4)	6 (3–9)	6 (4–9)	12 (8–15)	9 (6–12)	8 (5–13)	15 (11–19)
*Baseline patient characteristics*							
Pain intensity, median (IQR)	3 (1–4)	3 (2–5)	2 (1–4)	4 (3–6)	4 (3–6)	4 (3–5)	5 (4–6)
*Proportion of patients with positive change score*^*b*^*, %*							
NDI	43	30	23	31	22	21	13
Örebro screening questionnaire	40	27	29	23	19	13	29
HSCL-10	25	20	31	29	20	13	18
Recovery expectation	63	52	31	26	38	30	26

Pain intensity from NRS: the 11-point numerical rating scale [24], Recovery expectations from “In your view, how large is the risk that your current pain may become persistent?” (0–10, 0 = no risk, 10 = very large risk) and psychosocial risk factors: Örebro Screening Questionnaire (0–100) [29, 33], HSCL-10: Hopkins Symptom Checklist measuring emotional stress (0–4) [28], NDI: Neck Disability Index (0–50) measuring disability [26], Health status (0–100) [58]

^a^Presented as median of individual mean pain intensity

^b^Positive change score: patients in the cohort’s 80th percentile for change in score between baseline and 1-year

### Visual trajectory selection and last quarter SMS-based classification

**Fig. 5:**
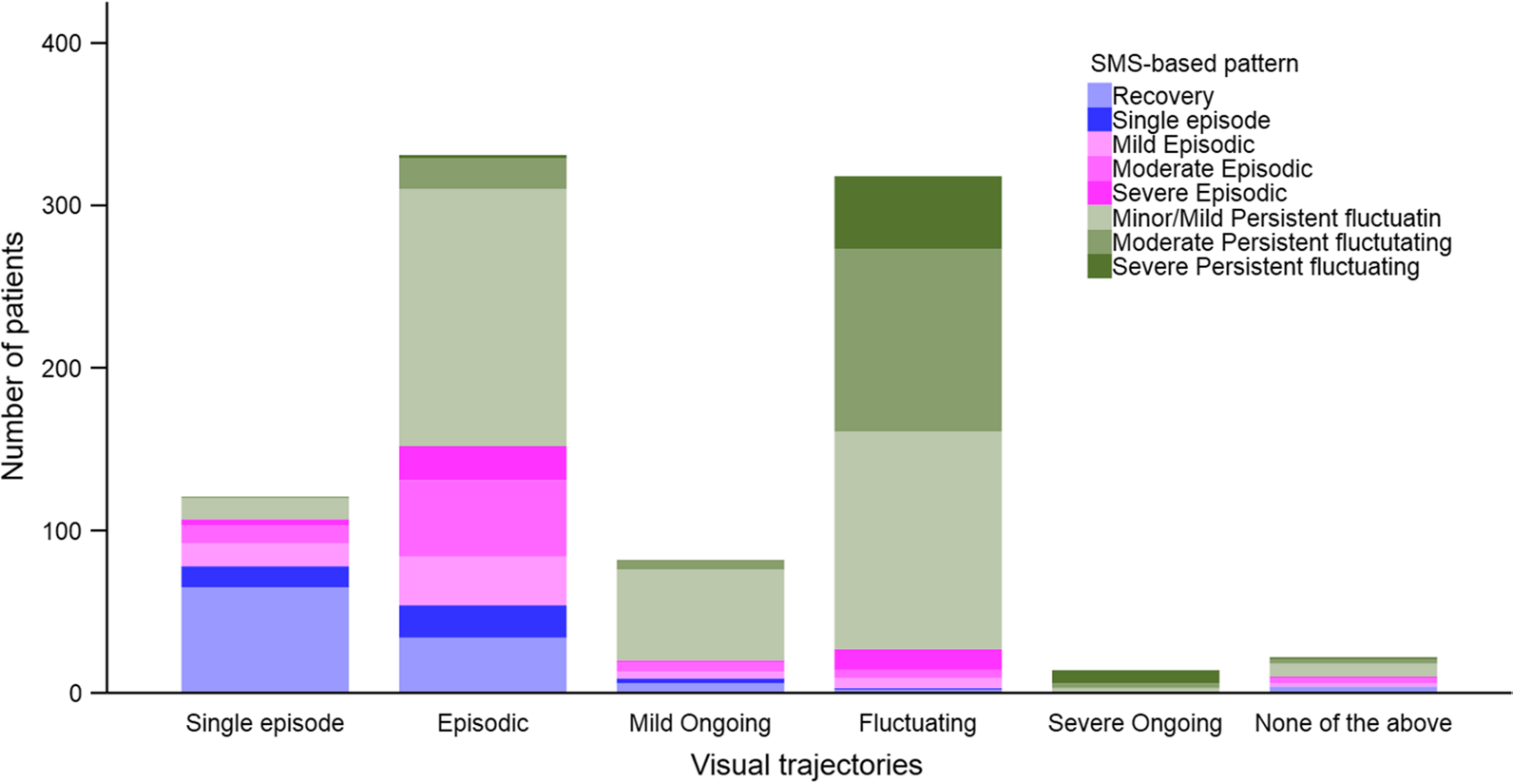
Relationship between the five visual trajectories and the last quarter SMS-based patterns

The main differences between comparing visual trajectories to the last quarter instead of the 1-year SMS-based classifications was those patients selecting Single episode visual trajectory were more often recovered in the last quarter but had reported episodes of pain previously during the full year (Fig. 5, for details see Additional file 1: Table S3). Also, most (77%) of the patients selecting the Mild ongoing visual trajectory were classified as Persistent based on SMS data in the last quarter, whereas this was the case for only 57% when considering the full year (see Additional file 1: Tables S2 and S3). In contrast, 68% of patients selecting the Episodic visual trajectory were classified as Episodic using SMS during the full year, but only 30% were classified as SMS-based Episodic in the last quarter.

Only 18% of the patients selecting the single episode visual trajectory were classified as Single episode/recovery for the full year (Fig. 4). However, all of them had their single episode in the last quarter (Table 3).

**Table 3:** Visual trajectory and SMS-based classification for 1-year versus last quarter

Visual trajectory	1-year SMS-based pattern	Last quarter SMS-based pattern, n (%)			
		Single episode/recovery	Episodic	Persistent fluctuating	Total
Single episode	Single episode/recovery	22 (100)	0	0	22
	Episodic	56 (62)	29 (32)	**6 (6)**	91
	Persistent fluctuating	0	0	8 (100)	8
Episodic	Single episode/recovery	2 (100)	0	0	2
	Episodic	52 (23)	97 (43)	**76 (34)**	225
	Persistent fluctuating	0	1 (1)	103 (99)	104
Mild ongoing	Single episode/recovery	0	0	0	0
	Episodic	9 (26)	11 (31)	**15 (43)**	35
	Persistent fluctuating	0	0	47 (100)	47
Fluctuating	Single episode/recovery	0	0	0	0
	Episodic	3 (5)	24 (37)	**38 (58)**	65
	Persistent fluctuating	0	0	253 (100)	253
Severe ongoing	Single episode/recovery	0	0	0	0
	Episodic	0	0	0	0
	Persistent fluctuating	0	0	14 (100)	14
Neither	Single episode/recovery	2 (100)	0	0	2
	Episodic	2 (20)	6 (60)	2 (20)	10
	Persistent fluctuating	0	0	10 (100)	10

Patients with a 1-year episodic SMS-based pattern and last quarter persistent fluctuating SMS-based pattern are marked in bold

Of the patients selecting Single episode visual trajectory (n = 121) and classified as Episodic SMS-based pattern for the full year, 62% were classified as Single episode in the last quarter. For patients classified as Episodic for the full year, there was an increase in the number classified as Persistent fluctuating in the last quarter (going from Single episode to Fluctuating visual trajectory).

## Discussion

The visual trajectories reflected the descriptors of the clinical course of pain captured by weekly SMS measures on a group level. Patients seemed to a large extend to recall both the pain variation and intensity dimensions of their neck pain. Patients' selection of the visual trajectories also appears to form groups that differ on other symptoms and patient characteristics. However, there were large variations in symptoms and characteristics within each visual trajectory and overlap rather than leaps between the trajectories. Thus, we cannot at this point conclude that the visual trajectories fully reflect the experienced course of NP. However, our results support that the visual trajectories and the SMS-based classifications are related on a group level.

### Clinical course and characteristics of patients in the different visual trajectories

Patients selecting Severe ongoing and Fluctuating visual trajectories reported the highest pain intensity and few to no pain-free weeks throughout the follow-up year. Hence, these two visual trajectories seem to be selected by patients with the highest disability and psychosocial risk factors, and with very low expectations of recovery. In addition, the large majority of these patients were classified as Persistent fluctuating throughout the follow-up year. However, we had only 14 patients selecting the Severe ongoing visual trajectory. They reported no pain-free weeks, more than half of their reported weeks were with severe pain, and all were classified as Persistent fluctuating. These are the only patients we can be certain had selected a visual trajectory that mostly reflected their clinical course. Still, a few of these patients reported some weeks with mild pain.

In contrast, only 18% of the patients selecting the Single episode visual trajectory actually reported only one single episode of pain during the 1-year follow-up (classified as Single episode/Recovery pattern). They typically reported several short episodes of pain on SMS throughout the follow-up year. Furthermore, one of these episodes most often occurred within the last quarter. One could thus hypothesize that recall bias plays a role in patients with few and short pain episodes, as such short episodes are less likely to be remembered over time [44]. Since these patients had high expectations of recovery and were mostly pain-free with negligible scores on symptoms and distress, one may suggest that their episodes are more tolerable and thus not easily recalled. The only comparable study, by Dunn et al. [20], had three visual trajectories representing the mildest clinical course options, illustrating single episode, few episodes and no or only little pain. These three trajectories were selected by patients having little pain and were negligibly affected. Thus, it is likely that the Single episode visual trajectory used in our study is sufficient to capture these patients with mild episodic pain and with minimal impact, even though most of them did not experience only one single episode. Even though our patients selecting Single episode visual trajectory typically have more than one single episode of pain during 1 year, it still is a group with a mild course of pain and little affliction.

The patients selecting the Episodic and Mild ongoing visual trajectories were comparable on most parameters, in particular: they reported mild to no pain most weeks, interspersed with flare-ups of pain that varied greatly in duration. The painful episodes also varied in intensity among patients in both visual trajectories, but weeks with severe pain were rarely reported. In addition, patients in both the Episodic and Mild ongoing visual trajectories scored moderate to low on all health-related factors. There are several possible explanations for these similarities. First, previous studies show that steady pain with minimal fluctuations is rare [13, 17], and a large group of patients with episodic pain report painful episodes lasting longer than 3 months [19]. Second, patients consider pain intensity ≤ 3 on NRS as a satisfactory state [45]. This could explain the similar patient characteristics in the Episodic and Mild ongoing visual trajectories, despite Episodic patients having had twice as many pain-free weeks as those selecting Mild ongoing. Third, some patients might simply not recall pain-free periods in a course mostly characterized by mild pain intensity, nor the duration of painful and pain-free periods [44, 46–48]. Nevertheless, the importance of periods with minor/no pain needs further examinations.

Thirty-eight percent of patients selected a visual trajectory that did not closely resemble their 1-year SMS-based classification. The visual trajectories are not simply a measure of pain, but more likely includes aspects of the pain experience, and have been shown to carry prognostic information as well as being related to expectation of pain [20–22]. The SMS-based classifications, on the other hand, are based on pain intensity measures and have a temporal aspect. Pain intensity is both subjective and complex, and thus likely not an adequate or complete measure of affliction related to pain [49–52]. It is generally accepted that pain scores are not easily compared between individuals. Moreover, recent studies have shown that pain intensity is not a good outcome measure compared to other health constructs [53]. It is therefore likely that the differences found between the visual trajectories and the SMS-based classification patterns reflects some of these factors.

### Strengths and weaknesses

The strength of this study is the large cohort and the good response rate. We have used descriptors and definitions for SMS-based patterns based on weekly measures over 1 year, which can easily be repeated based on previous published recommendations [5]. This has allowed us to identify the large variation in individuals’ course of pain over time, which are not found in studies that use two to three measurements during a 1-year follow-up time [54, 55]. In addition, we included patients with neck pain, regardless of the time of pain onset and treatment duration. It is therefore likely that our findings reflect a general distribution of the visual trajectories of neck pain patients in chiropractic practice. We included the options, “Do not know” and “Neither”, for responders who did not recognize any of the visual trajectories, and these answer-alternatives accounted for only 2% of our patients. It is therefore doubtful that we have missed relevant information regarding the understanding of the trajectories.

The weaknesses of the study are that the visual trajectories used have not been validated, and there are no studies for direct comparison. However, there is evidence of face, criterion, and construct validity of similar visual trajectories [20]. We did not include an extra visual trajectory questionnaire specifically for the last quarter. Hence, we can only hypothesize about recall bias and its effect on the selection of Single episode and Episodic trajectories, and these results must be interpreted with care. Furthermore, the differences in NDI (function), HSCL-10 (emotional distress) and Örebro (psychological risk factors) between the visual trajectories were often below proposed minimal clinical important differences, and conclusions regarding differences between the trajectories should be interpreted more as trends. Based on results from previous studies using latent class analyses [5, 14, 15], Dunn et al. included two visual trajectories “Gradual improvement” and “Gradual worsening” [20]. Even though these were selected by only 5% (improvement) and 4% (worsening) of their patients, we cannot exclude that these might be relevant for neck pain patients in chiropractic care. Lastly, since 33% of the cohort was lost to follow-up, we cannot exclude that this has introduced bias in the results. However, we find this within an acceptable range considering the type of study and duration of follow-up [56].

### Clinical implications and future indications

In clinical practice, the visual trajectories are likely more applicable than frequent measures over time. The visual trajectories can be useful as a communication tool between patient and clinician regarding the course and prognosis of neck pain. They are simple to implement and seem easy to understand for patients and clinicians. The visual trajectories can potentially be used in clinic as a measure of pain history, but also as a picture of patients’ condition and illness perception here and now. Patients with similar observed clinical course have different recall of their neck pain experience, and it would be of interest to understand more regarding the factors that influence this difference in recollection. Based on our study and a very recent study showing that similar visual trajectories are relatively stable over time [57], the visual trajectories have potential for use in prognostic research. Both as a substitute to frequent measures, and in combination with other factors in prediction models and phenotypes for prediction and/or subgrouping.

However, our descriptive study indicates that both the visual trajectory pattern questionnaire and the SMS-based pattern definitions need more refinement. Patients classified as Episodic pattern but selected the Single episode visual trajectory reported more weeks with minor pain, longer pain-free periods, and were generally less afflicted by their pain than those selecting the Episodic visual trajectory. Furthermore, patients selecting Single episode visual trajectory but classified as Episodic pattern closely resembled the patients selecting the Single episode visual trajectory. Consequently, the text to the Single episode might be more appropriate, and more easily understood, if the wording was changed to “No neck pain or just very short episodes of neck pain”, instead of the present “…just a single episode…”. Also, it is likely that combining some of the illustrations, for instance Episodic and Mild ongoing visual trajectory, would be beneficial.

Future studies should explore the differences between patients selecting a visual trajectory that closely matches their SMS-based clinical course pattern, and patients selecting visual trajectories more “positive” or more “negative” than their classified pattern.

## Conclusions

The visual trajectories used in this study generally reflect the patients’ clinical course defined by SMS data on group level. However, it is not a perfect match. This can be due to recall bias, but just as likely, that a patient’s experienced course of pain is not based on pain intensity alone. Our findings suggest that the visual trajectories and SMS-based classifications may capture different elements of the pain experience. The visual trajectories most likely represent pictures that encompass features of the patients’ course of pain, individual level of pain tolerance, and clinical condition at the time of reporting. Therefore, they cannot be seen as a proxy for SMS-tracking of pain intensity over 1 year. Rather, visual trajectories may be a suitable tool to attain a broader picture for prediction of NP or stratification of NP patients.

## Supplementary Information

**Additional file 1.** Imputation of the weekly SMS data before classification and Classification into patterns and subgroups. **Table S1** Definitions of the SMS-based patterns. **Table S2** Association between the Visual trajectories and the 1-year SMS-based patterns (n = 888). **Table S3** Association between the Visual trajectories and the last quarter SMS-based patterns (n = 888)

## Data Availability

The datasets generated and/or analyzed during the current study are not publicly available due to data protection policies but are available from the corresponding author on reasonable request.

## Abbreviations

SMS: Short messaging services
NRS: Numeric rating scale
NDI: Neck disability index
HSCL-10: Hopkins symptom checklist
NPQ: Nordic pain questionnaire
MIC: Minimal important change
SD: Standard deviation
IQR: Inter-quartile range
NP: Neck pain
