# Supplementary material for: Exploring visual pain trajectories in neck pain patients, using clinical course, SMS-based patterns, and patient characteristics: a cohort study
Source: Chiropr Man Therap. 2022 Sep 8;30:37. doi: 10.1186/s12998-022-00443-3 (PMC9454174; doi:10.1186/s12998-022-00443-3)
Supplement: Supplementary file 1 — Additional file 1. Imputation of the weekly SMS data before classification and Classification into patterns and subgroups. Table S1 Definitions of the SMS-based patterns. Table S2 Association between the Visual trajectories and the 1-year SMS-based patterns (n = 888). Table S3 Association between the Visual trajectories and the last quarter SMS-based patterns (n = 888) [file 12998_2022_443_MOESM1_ESM.docx]

**Additional file 1**

**Supplementary file 1**

*Imputation of the weekly SMS data before classification*

We imputated missing values on the weekly pain intensity measures from SMS in three stages as follows: (1) we replaced missing responses in week 52 by the values reported in week 51, (2) one-week and two-week gaps between weeks with the same pain intensity, were replaced with that same value; (3) patients who after steps 1 and 2 had less than 26 complete responses out of 52 were excluded from the analysis and categorized as missing.

*Classification into patterns and subgroups*

The original definitions used in this study were based on conclusions drawn from a collaborative group on the clinical course of LBP (Kongsted et al., 2016), and translated into four variation patterns and tested on a LBP cohort (Kongsted et al., 2017): Ongoing, Fluctuating, Episodic and Single episode patterns. Patients in the Ongoing pattern should have a variation in pain intensity that did not exceed ±1 from the mean value each week. For the Fluctuating pattern, patients should have no consecutive four-week or more pain-free, and variation from the mean pain had to exceed ±1. Patients in the Episodic pattern should have pain-free periods of minimum four consecutive weeks between periods with pain. The definition of an episode was based on previously suggested definitions by de Vet et al (de Vet et al., 2002) and later upheld by a Delphi study (Stanton et al., 2011) and tested on a LBP cohort (Eklund et al., 2016), where an episode of LBP is defined as a period of pain lasting more than 24 hours, preceded and followed by at least four pain free weeks. Patients with a Single episode could have only one episode lasting 1-2 weeks during the study period. In addition, the Single episode could not be at the end of the study period. Findings and subsequent suggestions from two previous studies using the original definitions (Irgens et al., 2020; Kongsted et al., 2017) resulted in the following alterations in the patterns. The two studies found that very few patients fit into the Ongoing and Single episode variation patterns. Also, Ongoing and Fluctuating patterns were very similar, where persistent pain was rarely completely steady in pain intensity (Irgens et al., 2020). We therefore combined the Ongoing and Fluctuating variation patterns into one pattern called Persistent pattern. In addition, patients classified in the Minor subgroups of Ongoing, Episodic and Single episode shared similar demographic, psychosocial and functional characteristics. These patients were only negligibly affected by their pain, and their pain intensity was also below what is considered clinically significant (<2 on NRS) (Kovacs et al., 2008; Pool et al., 2007). As a result, all patients with maximum pain intensity <2 were included in a new pattern called Recovery. However, patients in the Minor Persistent fluctuating subgroup were significantly more affected by their pain than the other three minor subgroups and were not included in the Recovery pattern. Details of the definitions of each pattern used in this study are shown in Supplementary table 1.

**Supplementary table 1** *Definitions of the SMS-based patterns*

| **Pattern label** | | **Variation pattern** | **Intensity** |
| --- | --- | --- | --- |
| **Recovery** | Recovery | Pain that is either Episodic or Single episode, or where mean intensity equals zero | **Maximum** intensity <2 |
| **Single episode** | Severe single episode | One episode lasting 1 - 2 weeks (which are not the first or the last week of measurement) | **Maximum** intensity ≥6  No restriction on mean intensity |
|  | Moderate single episode | One episode lasting 1 - 2 weeks (which are not the first or the last week of measurement) | **Maximum** intensity ≥4 and <6  No restriction on mean intensity |
|  | Mild single episode | One episode lasting 1 - 2 weeks (which are not the first or the last week of measurement) | **Maximum** intensity ≥2 and <4  No restriction on mean intensity |
| **Episodic** | Severe episodic | Pain-free periods of min. 4 weeks in a row between weeks with pain. Four weeks or more without pain in the beginning or end of the course does not indicate a new episode. | **Maximum** intensity ≥6  No restriction on mean intensity |
|  | Moderate episodic | Pain-free periods of min. 4 weeks in a row between weeks with pain. Four weeks or more without pain in the beginning or end of the course does not indicate a new episode. | **Maximum** intensity ≥4 and <6  No restriction on mean intensity |
|  | Mild episodic | Pain-free periods of min. 4 weeks in a row between weeks with pain. Four weeks or more without pain in the beginning or end of the course does not indicate a new episode. | **Maximum** intensity ≥2 and <4  No restriction on mean intensity |
| **Persistent fluctuating** | Severe persistent | No pain-free 4-weeks periods | **Mean** intensity ≥6  No restrictions on maximum intensity |
|  | Moderate persistent | No pain-free 4-weeks periods | **Mean** intensity ≥4 and <6  No restrictions on maximum intensity |
|  | Mild persistent | No pain-free 4-weeks periods | **Mean** intensity ≥2 and <4  No restrictions on maximum intensity |
|  | Minor persistent | No pain-free 4-weeks periods | **Mean** intensity <2  No restrictions on maximum intensity |

**Supplementary table 2** Association between the Visual trajectories and the 1-year SMS-based patterns (n=888)

|  |  | **1-year SMS-based pattern distribution, (n%)** | | | | | | | | | | |  |
| --- | --- | --- | --- | --- | --- | --- | --- | --- | --- | --- | --- | --- | --- |
|  |  | **Recovery** | **Single episode** | | | **Episodic** | | | **Persistent fluctuating** | | | |  |
| **Visual trajectory** |  |  | Severe | Moderate | Mild | Severe | Moderate | Mild | Severe | Moderate | Mild | Minor | **Total** |
| **Single episode** |  | 14 (12) | 2 (2) | 0 | 6 (5) | 36 (30) | 37 (31) | 18 (15) | 0 | 0 | 7 (6) | 1 (1) | 122 (14) |
| **Episodic** |  | 1 (<1) | 0 | 0 | 1 (<1) | 143 (43) | 61(18) | 21(6) | 1 (<1) | 17 (5) | 73 (22) | 13 (4) | 331 (37) |
| **Mild ongoing** |  | 0 | 0 | 0 | 0 | 20 (24) | 12 (15) | 3 (4) | 0 | 7 (9) | 32 (39) | 8 (10) | 82 (9) |
| **Fluctuating** |  | 0 | 0 | 0 | 0 | 59 (19) | 6 (2) | 0 | 30 (9) | 113 (36) | 105 (33) | 5 (2) | 318 (36) |
| **Severe ongoing** |  | 0 | 0 | 0 | 0 | 0 | 0 | 0 | 7 (50) | 6 (43) | 1 (7) | 0 | 14 (2) |
| **Neither** |  | 0 | 0 | 0 | 2 (9) | 6 (27) | 3 (14) | 1 (5) | 1 (5) | 4 (18) | 5 (23) | 0 (0) | 22 (2) |
| **Total** |  | 15 (2) | 2 (<1) | 0 | 9 (1) | 264 (30) | 119 (13) | 43 (5) | 39 (4) | 147 (17) | 223 (25) | 27 (3) | 888 (100) |

**Supplementary 3able 3** Association between the Visual trajectories and the last quarter SMS-based patterns (n=888)

|  |  | **1-year SMS-based pattern distribution, (n%)** | | | | | | | | | | |  |
| --- | --- | --- | --- | --- | --- | --- | --- | --- | --- | --- | --- | --- | --- |
|  |  | **Recovery** | **Single episode** | | | **Episodic** | | | **Persistent fluctuating** | | | |  |
| **Visual trajectory** |  |  | Severe | Moderate | Mild | Severe | Moderate | Mild | Severe | Moderate | Mild | Minor | **Total** |
| **Single episode** |  | 65 (53) | 0 | 1 (1) | 12 (10) | 4 (4) | 11 (9) | 14 (12) | 0 | 1 (1) | 5 (4) | 8 (7) | 122 (14) |
| **Episodic** |  | 34 (10) | 4 (1) | 7 (2) | 9 (3) | 21 (6) | 47 (14) | 30 (9) | 2 (1) | 19 (6) | 106 (32) | 52 (16) | 331 (37) |
| **Mild ongoing** |  | 6 (7) | 1 (1) | 1 (1) | 1 (1) | 1 (1) | 6 (7) | 4 (5) | 0 | 6 (7) | 36 (44) | 20 (24) | 82 (9) |
| **Fluctuating** |  | 2 (1) | 1 (<1) | 0 | 0 | 13 (4) | 5 (2) | 6 (2) | 45 (14) | 112 (35) | 111 (35) | 23 (7) | 318 (36) |
| **Severe ongoing** |  | 0 | 0 | 0 | 0 | 0 | 0 | 0 | 8 (57) | 3 (21) | 3 (21) | 0 | 14 (2) |
| **Neither** |  | 4 (18) | 0 | 0 | 0 | 1 (5) | 3 (14) | 2 (9) | 1 (5) | 3 (14) | 6 (27) | 2 (9) | 22 (2) |
| **Total** |  | 111 (12) | 6 (1) | 9 (1) | 22 (2) | 41 (5) | 72 (8) | 56 (6) | 56 (6) | 144 (16) | 267 (30) | 105 (12) | 888 (100) |

**References**

Boonstra, A. M., Schiphorst Preuper, H. R., Balk, G. A., & Stewart, R. E. (2014). Cut-off points for mild, moderate, and severe pain on the visual analogue scale for pain in patients with chronic musculoskeletal pain. *Pain*, *155*(12), 2545-2550. <https://doi.org/10.1016/j.pain.2014.09.014>

Boonstra, A. M., Stewart, R. E., Köke, A. J., Oosterwijk, R. F., Swaan, J. L., Schreurs, K. M., & Schiphorst Preuper, H. R. (2016). Cut-Off Points for Mild, Moderate, and Severe Pain on the Numeric Rating Scale for Pain in Patients with Chronic Musculoskeletal Pain: Variability and Influence of Sex and Catastrophizing. *Frontiers in Psychology*, *7*, 1466. <https://doi.org/10.3389/fpsyg.2016.01466>

de Vet, H. C., Heymans, M. W., Dunn, K. M., Pope, D. P., van der Beek, A. J., Macfarlane, G. J., Bouter, L. M., & Croft, P. R. (2002). Episodes of low back pain: a proposal for uniform definitions to be used in research. *Spine (Phila Pa 1976)*, *27*(21), 2409-2416. <https://doi.org/10.1097/01.brs.0000030307.34002.be>

Eklund, A., Jensen, I., Lohela-Karlsson, M., Leboeuf-Yde, C., & Axen, I. (2016). Absence of low back pain to demarcate an episode: a prospective multicentre study in primary care. *Chiropr Man Therap*, *24*, 3. <https://doi.org/10.1186/s12998-016-0085-z>

Fejer, R., Jordan, A., & Hartvigsen, J. (2005). Categorising the severity of neck pain: Establishment of cut-points for use in clinical and epidemiological research. *Pain*, *119*(1-3), 176-182. <https://doi.org/http://dx.doi.org/10.1016/j.pain.2005.09.033>

Irgens, P., Kongsted, A., Myhrvold, B. L., Waagan, K., Engebretsen, K. B., Natvig, B., Vollestad, N. K., & Robinson, H. S. (2020). Neck pain patterns and subgrouping based on weekly SMS-derived trajectories. *BMC Musculoskelet Disord*, *21*(1), 678. <https://doi.org/10.1186/s12891-020-03660-0>

Kongsted, A., Hestbaek, L., & Kent, P. (2017). How can latent trajectories of back pain be translated into defined subgroups? *BMC Musculoskelet Disord*, *18*(1), 285. <https://doi.org/10.1186/s12891-017-1644-8>

Kongsted, A., Kent, P., Axen, I., Downie, A. S., & Dunn, K. M. (2016). What have we learned from ten years of trajectory research in low back pain? *BMC Musculoskelet Disord*, *17*, 220. <https://doi.org/10.1186/s12891-016-1071-2>

Kovacs, F. M., Abraira, V., Royuela, A., Corcoll, J., Alegre, L., Tomas, M., Mir, M. A., Cano, A., Muriel, A., Zamora, J., Del Real, M. T., Gestoso, M., Mufraggi, N., & Spanish Back Pain Research, N. (2008). Minimum detectable and minimal clinically important changes for pain in patients with nonspecific neck pain. *BMC Musculoskelet Disord*, *9*, 43. <https://doi.org/10.1186/1471-2474-9-43>

Pool, J. J. M., Ostelo, R. W. J. G., Hoving, J. L., Bouter, L. M., & De Vet, H. C. W. (2007). Minimal clinically important change of the neck disability index and the numerical rating scale for patients with neck pain. *Spine (Phila Pa 1976)*, *32*(26), 3047-3051. <https://doi.org/http://dx.doi.org/10.1097/BRS.0b013e31815cf75b>

Serlin, R. C., Mendoza, T. R., Nakamura, Y., Edwards, K. R., & Cleeland, C. S. (1995). When is cancer pain mild, moderate or severe? Grading pain severity by its interference with function. *Pain*, *61*(2), 277-284. <https://doi.org/10.1016/0304-3959(94)00178-h>

Stanton, T. R., Latimer, J., Maher, C. G., & Hancock, M. J. (2011). A modified Delphi approach to standardize low back pain recurrence terminology. *European Spine Journal*, *20*(5), 744-752. <https://doi.org/10.1007/s00586-010-1671-8>
